# Relapsing Polychondritis

**DOI:** 10.31662/jmaj.2019-0045

**Published:** 2019-10-10

**Authors:** Daisuke Taniyama, Taketomo Maruki, Tetsuya Sakai

**Affiliations:** 1 Department of General Internal Medicine, Tokyo Saiseikai Central Hospital, Tokyo, Japan; 2 Department of Thoracic Oncology, National Cancer Center Hospital East, Chiba, Japan

**Keywords:** relapsing polychondritis, bronchoscopy, airway obstruction

A 61-year-old man with a history of sinusitis was admitted due to fever, pain in the left side of the nose, and arthralgia. Clinical examination showed tenderness and swelling of the left nasal cartilage, left knee joint, and left sternoclavicular joint. Contrast-enhanced computed tomography (CT) was performed on the next day because he developed cough and dyspnea. CT revealed thickening of the tracheal and bilateral bronchial tube walls (Figure 1a, b); bronchoscopy showed remarkable redness of the bronchial mucosa, and white lesions were observed except in the membranous portion (Figure 2). Tracheal biopsy revealed acute inflammation (Figure 3a, b). Relapsing polychondritis (RP) was diagnosed, and prednisolone (1 mg/kg; 60 mg/body) was administered. Consequently, his symptoms were resolved, and the laryngo-tracheobronchial luminal narrowing and wall thickening improved. RP is an autoimmune disease characterized by inflammation and destruction of cartilaginous tissues and can affect several organs. RP complicated by laryngo-tracheobronchial disease may lead to airway obstruction, resulting in even death ^(1)^.

**Figure 1 a, b.**
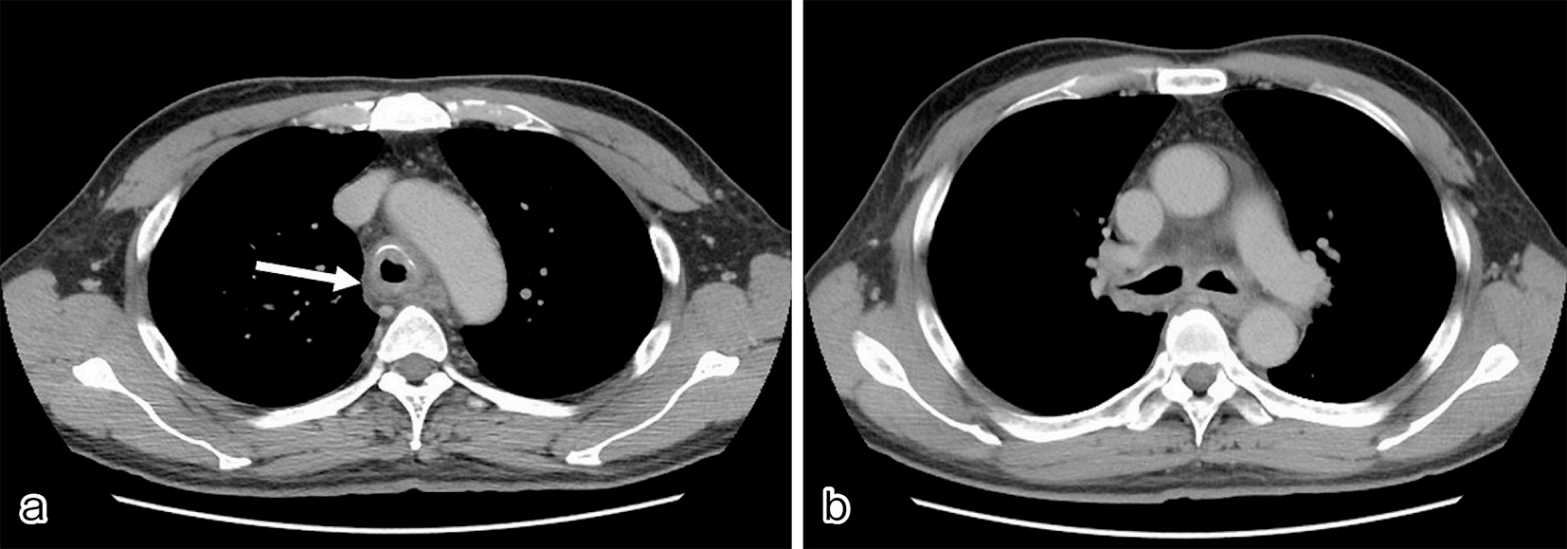
Contrast-enhanced computed tomography revealed thickening of the tracheal and bilateral bronchial tube walls.

**Figure 2.**
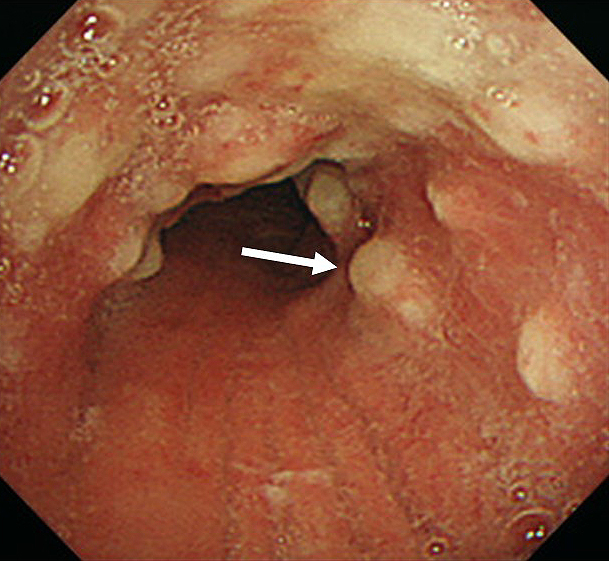
Bronchoscopy showed remarkable redness of the bronchial mucosa, and white lesions were observed except in the membranous portion.

**Figure 3 a, b.**
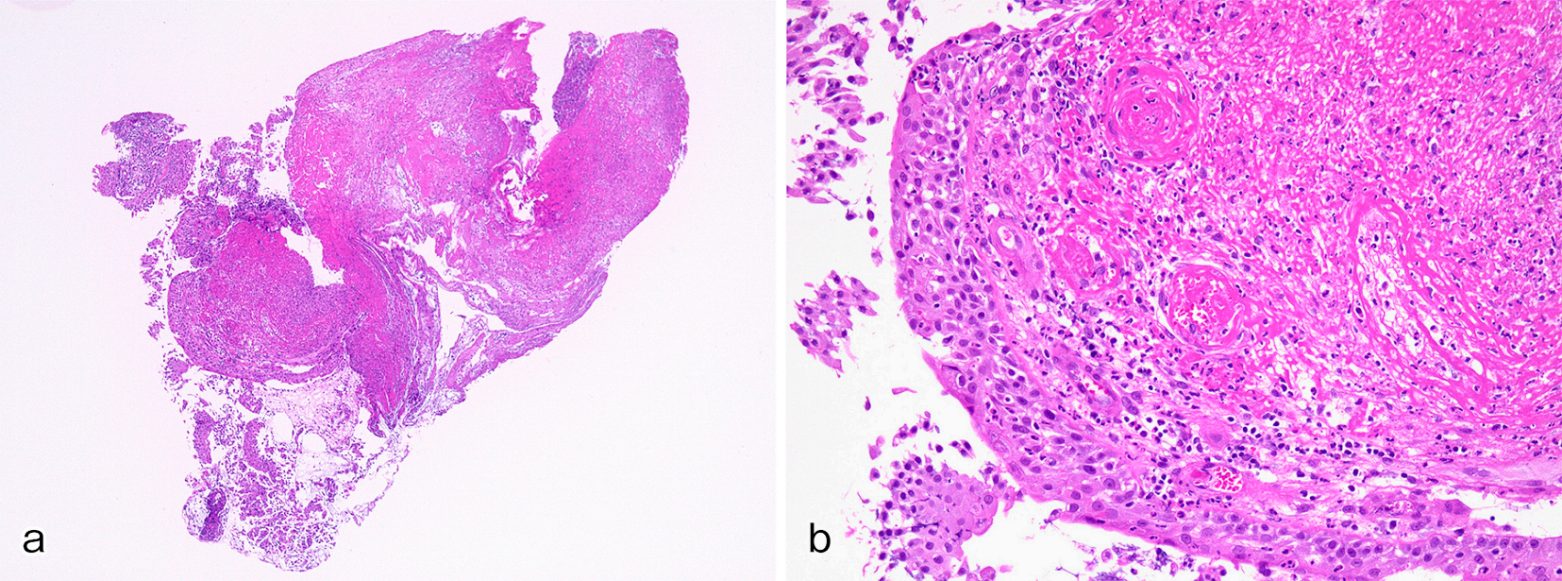
Tracheal biopsy revealed acute infiltration of various inflammatory cells mainly composed of neutrophils and eosinophils in the tracheal mucosa and inflammatory exudate precipitation and myxomatous change in the subepithelial tissue, compatible with relapsing polychondritis in low-power field (a) and in high-power field (b).

## Article Information

### Conflicts of Interest

None

### Author Contributions

Daisuke Taniyama, Taketomo Maruki, and Tetsuya Sakai contributed to the report concept and design.

Daisuke Taniyama and Tetsuya Sakai performed the acquisition of patient’s data.

Daisuke Taniyama prepared and wrote the manuscript.

### Informed Consent

Informed consent has been obtained from the patient to publish the information, including their photographs.

### Approval by Institutional Review Board (IRB)

In this study, IRB approval was not required.
